# Untargeted blood serum proteomics identifies novel proteins related to neurological recovery after human spinal cord injury

**DOI:** 10.1186/s12967-024-05344-y

**Published:** 2024-07-17

**Authors:** Daniel Garcia-Ovejero, Evelyn Beyerer, Orpheus Mach, Iris Leister, Martin Strowitzki, Christof Wutte, Doris Maier, John LK Kramer, Ludwig Aigner, Angel Arevalo-Martin, Lukas Grassner

**Affiliations:** 1 Laboratory of Neuroinflammation, Hospital Nacional de Paraplejicos, SESCAM, Toledo, Spain; 2 Institute of Molecular Regenerative Medicine, Paracelsus Medical University, Salzburg, Austria; 3 Spinal Cord Injury Center, BG Trauma Center, Murnau, Germany; 4 ParaMove, SCI Research Unit, BG Tauma Center Murnau, Germany and Paracelsus Medical University Salzburg, Salzburg, Austria; 5 Department of Neurosurgery, BG Trauma Center, Murnau, Germany; 6 International Collaboration on Repair Discoveries, ICORD, University of British Columbia, Vancouver, Canada; 7 Department of Anesthesiology, Pharmacology & Therapeutics, Faculty of Medicine, University of British Columbia, Vancouver, Canada; 8 Department of Neurosurgery, Christian Doppler Clinic, Paracelsus Medical University, Salzburg, Austria

**Keywords:** SERPINE1, RAGE, Calumenin, CD300, ARHGAP35, Defensin, SCI, Thromboinflammation, Coagulation, Inflammation

## Abstract

**Background:**

The discovery of new prognostic biomarkers following spinal cord injury (SCI) is a rapidly growing field that could help uncover the underlying pathological mechanisms of SCI and aid in the development of new therapies. To date, this search has largely focused on the initial days after the lesion. However, during the subacute stage of SCI (weeks to months after the injury), there remains potential for sensorimotor recovery, and numerous secondary events develop in various organs. Additionally, the confounding effects of early interventions after the injury are less likely to interfere with the results.

**Methods:**

In this study, we conducted an untargeted proteomics analysis to identify biomarkers of recovery in blood serum samples during the subacute phase of SCI patients, comparing those with strong recovery to those with no recovery between 30 and 120 days. We analyzed the fraction of serum that is depleted of the most abundant proteins to unmask proteins that would otherwise go undetected. Linear models were used to identify peptides and proteins related to neurological recovery and we validated changes in some of these proteins using Enzyme-linked Immunosorbent Assay (ELISA).

**Results:**

Our findings reveal that differences in subacute recovery after SCI (from 30 to 120 days) are associated with an enrichment in proteins involved in inflammation, coagulation, and lipid metabolism. Technical validation using commercial ELISAs further confirms that high levels of SERPINE1 and ARHGAP35 are associated with strong neurological recovery, while high levels of CD300a and DEFA1 are associated with a lack of recovery.

**Conclusions:**

Our study identifies new candidates for biomarkers of neurological recovery and for novel therapeutic targets after SCI.

**Supplementary Information:**

The online version contains supplementary material available at 10.1186/s12967-024-05344-y.

## Background

In spinal cord injury (SCI), parameters that support accurate diagnosis, predict the clinical outcome, evaluate response to treatments, or, ideally, enlighten mechanisms of damage that may lead to new therapeutic tools for recovery, are largely missing. Such parameters may be considered “biomarkers” and comprise, among others, proteins, immune system mediators, cell populations, transcriptomes of specific cell types, routine blood measurements, or imaging features [1–12].

To date, the search for biomarkers after SCI has been largely focused on the first days after lesion, with the aim to establish a realistic prognosis of recovery and tailor the most effective therapeutical strategy with each patient [1, 2, 5, 6]. Pathological processes at this time evolve dynamically, imposing narrow useful time windows to detect some markers, as occurs with the detection of neural-derived proteins in blood [10, 13]. In addition, pharmacological management at the very acute and acute phases may interfere with processes suitable of render biomarkers of recovery. On the other hand, in the subacute stage of SCI (from weeks to months after injury), there is still potential for sensorimotor recovery, numerous secondary events develop in various organs and patients are stabilized [1, 11, 14].

Biomarkers can be obtained from different possible sources, but an advantage of blood-derived biomarkers is the easy sampling and handling by making use of standard clinical procedures [15]. In addition, they offer information about the physiological or pathological status of many organs, which may be relevant in a pathology like SCI that causes multi-organ dysfunction [16–20]. So far, non-supervised searches for protein markers in the blood faced a common problem: the heterogeneity of blood proteins concentration renders a proteome with a high dynamic range, in which a low number of high abundant proteins (HAPs) may account for 99% of the total protein mass [21, 22]. For instance, albumin can be found at mg/ml levels whereas other proteins (LAPs, for “Lower Abundant Proteins”) are found in ng/ml or pg/ml ranges [21, 22]. Several HAPs have been identified as biomarkers after SCI both by hypothesis-driven and untargeted (hypothesis-free) strategies [1, 2, 5, 23], while LAPs have been explored as biomarkers only in hypothesis-driven strategies (searching for previously selected proteins) [1, 5, 24, 25]. Here, we used untargeted proteomics to identify new biomarker candidates unmasked after depletion of HAPs in patients blood sera.

Using this strategy, we found proteins differentially expressed in patients with strong sensorimotor recovery versus those with no recovery. Further, we highlight biological processes and molecular pathways involved in these differences between both groups. We also validated some of these proteins using routine assay techniques (Enzyme-linked Immunosorbent Assay; ELISA) that might be easily adopted in clinical laboratories.

## Methods

### Patients and healthy control subjects

A cohort of thirty patients with motor complete (American Spinal Injury Impairment Scale -AIS- A and B) traumatic spinal cord injury (SCI) was selected among those recruited at the Trauma Center Murnau (Bavaria, Germany) or at Hospital Nacional de Paraplejicos (Toledo, Spain) under the development of the Autoantibodies in Spinal Cord Injury study [26] (Table 1). Blood samples included in this study were collected during the subacute phase (31 ± 1 days post-injury). The study was approved by the Ethics Committee of Toledo Health Care Area and by the Ethics Committee of the Bavarian Medical Board (registry number 15,046). The study follows and adheres to the World Medical Association Declaration of Helsinki and is registered at the public database Clinicaltrial.gov (NCT02493543). All patients fulfilled the inclusion and exclusion criteria and gave their informed consent to participate.

**Table 1:** Clinical and demographical characteristics of patients with spinal cord injury

	Strong Recovery (SR)	No Recovery (NR)
Age (years) Range Mean ± sem	19–79 37.1 ± 13.4	18–74 40 ± 7.8
Sex (n) Female Male	4 6	5 15
Lesion level (n) Tetraplegia Paraplegia	6 4	12 8
AIS grade (n) A B	2 8	16 4

Inclusion criteria were:

- Males and females.
- At least 18 years old.
- Any neurological level of injury, except cauda equina syndrome.
- Complete and incomplete lesions.
- If patient was treated with glucocorticoids, the last dose should have been.

administered at least 7 days before study onset. Exclusion criteria were:

- Diagnosed autoimmune disorder.
- Diagnosed tumor.
- Pre-existing neurodegenerative disease.

In addition, patients with traumatic brain injury (Glasgow Coma Scale < 14) were excluded from this study. Polytrauma patients were recruited as long as their injuries did not interfere with neurological examination.

Sensorimotor function of patients was evaluated following the International Standards for Neurological Classification of Spinal Cord Injury scale (ISNCSCI) at an average time of 31 ± 1 days after injury (from now on described as 4 weeks after injury). All evaluations were performed by experienced personnel. At the same dates, a blood sample was obtained from each patient. Clinical and demographical characteristics of patients are summarized in Table 1.

For the ELISA studies, the levels of some proteins were compared with control individuals. Two different control groups were included: (i) Healthy control group (HC), formed by voluntary healthy individuals recruited at Murnau Trauma Hospital (*n* = 41; Supplementary Table S1) and (ii) Spine fracture control group (SPFC), formed by patients with spine fracture between C1 and L1 but no neurological damage, recruited at Murnau Trauma Hospital (*n* = 9; Supplementary Table S1). In the control groups, a single blood sample was taken after signing the informed consent and fulfilling the inclusion and exclusion criteria detailed above (with the obvious exception of not suffering a SCI). Age and sex of these individuals are summarized in Supplementary Table S1. Both HC and SPFC had not previous history of neurological trauma or neurological deficits. In addition, HC were asymptomatic at the time of recruitment.

**Fig. 1:**
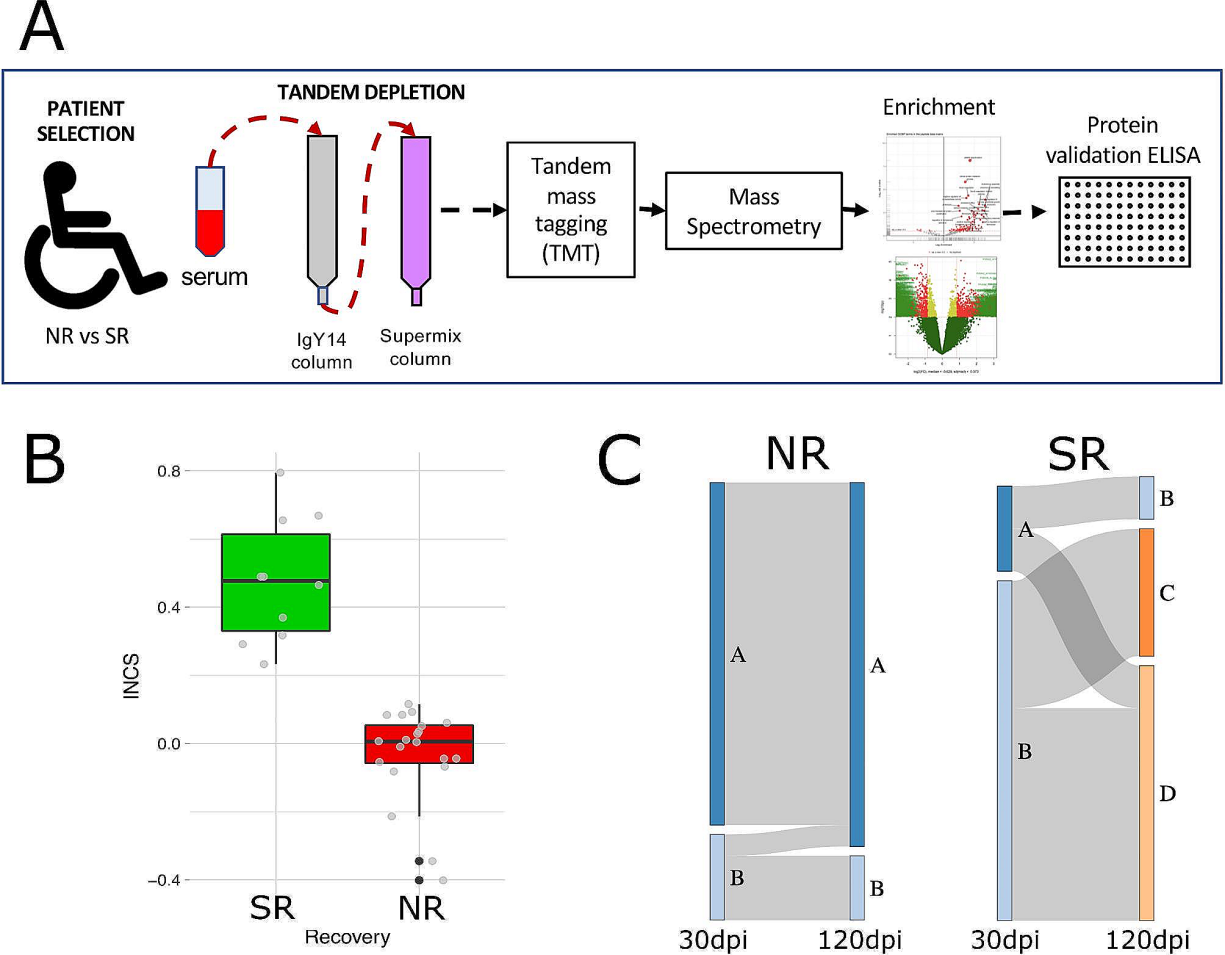
Experimental Design. **(A)** Scheme depicting the workflow used in this study. Sera were collected from SCI patients with no (NR) or strong (SR) recovery and depleted from high and medium abundant proteins for subsequent tagging and detection of low abundant proteins using mass spectrometry. After enrichment analysis (NR vs. SR), some of these proteins were further validated by ELISA. **(B)** Box plot showing the distribution of SR (green) and NR (red) patients studied according to their Integrated Neurological Change Score (INCS) between 30 and 120 days after injury. **(C)** Distribution of SR (green) and NR (red) patients according to their AIS grade conversion (-1 to + 3) between 30 and 120 days after injury

A scheme of the workflow followed in the current study can be found in Fig. 1.

### Patient stratification: strong vs. no-recovery

Patients were classified as showing no recovery or strong recovery based on AIS grade conversion and INCS (Integrated Neurological Change Score; [27]), a score of overall change in the neurological function of patients assessed according to the International Standards for the Neurological Classification of Spinal Cord Injury (ISNCSCI; [28]). Patients who did not convert their AIS grade from 30 to 120 days post-injury (dpi) and who showed INCS values (for the same period) close to zero or negative –did not experience significant changes in the overall neurological function or even experienced some worsening– were classified as no recovery (NR; Fig. 1B, C). Patients with AIS grade conversion and INCS values significantly higher than those of the previous group were classified as strong recovery (SR; Fig. 1B, C). Indeed, median INCS value of SR group is close to 0.5, which may be interpreted as recovering at 120 dpi half of the overall neurological function that was not present at 30 dpi (Fig. 1B). Based on these criteria, 10 patients were classified as SR and 20 as NR.

### Serum samples collection and processing

Peripheral blood was collected by venipuncture at the medial cubital vein. Blood clot was allowed to form by maintaining the tubes for 45 min at room temperature (RT) followed by 1 h at 4ºC. Blood was centrifuged at 4ºC, at 1500 g for 20 min and serum was aliquoted and stored at -80ºC until used.

A tandem IgY14/Supermix depletion method, following that described in Keshishian et al. [29], was used in this study. Serum volumes corresponding to a starting mass of ~ 12.5 mg were immuno-affinity- depleted of the 14 most abundant proteins followed by the next ~ 50 moderately abundant proteins using Seppro® IgY14 (LC10) and Seppro® Supermix (LC5) columns (both columns from Sigma- Aldrich, St. Louis, MO, USA). In an HPLC-assisted manner (Waters Alliance 2695), serum was first loaded on the IgY14 column and the flow-through directed onto the Supermix column. Dilution, stripping and neutralization buffers provided by the manufacturer were used and manufacturer’s instructions were followed (Sigma-Aldrich). Flow-through that included proteins based on UV absorbance was collected and concentrated by spin filters (Amicon 3 kDa MWCO; Millipore) to a volume of ~ 500 µl. The protein concentrations of the samples post-depletion were determined by Bradford protein assay. Furthermore, the protein content was visualized by Coomassie stained (Imperial Stain, Pierce) SDS-PAGE 4–20% gradient gels (Criterion, Biorad; Supplementary Figure S1). Collectively, this procedure yields a ~ 100-fold enrichment of LAPs and a reduction of, at least, two orders of magnitude in the dynamic range of protein concentrations, allowing LAPs to be detected by mass spectrometry [22, 30]. The proteins depleted by both columns are listed in Supplementary Table S2.

Serum depletion, tandem mass tagging (TMT) and mass spectrometry (including peptide and protein identification and scoring) were performed by Proteome Sciences (Proteome Sciences plc, Surrey, UK), and includes several quality control steps (detailed below).

### TMT labeling and Mass spectrometry (TMT MS2)

Depleted samples were combined into three tandem mass tags (TMT) 11plexes which were processed and analyzed by the TMT®MS2 workflow (Proteome Sciences, UK). The workflow was the following: in each TMT® 11plex, ten experimental samples were combined with one reference pool; the three TMT® 11plexes were separated using basic reverse phase (bRP) chromatography and 30 fractions collected; each fraction was subjected to LC-MS2 analysis using a high-performance Orbitrap Fusion mass spectrometer (Thermo Scientific).

More in detail, all samples were adjusted to the same protein concentration by adding depletion’s dilution buffer (Sigma). Equal volumes from all samples were taken to prepare the pooled reference sample. Volumes equivalent to 50 µg of protein per sample were reduced (dithiothreitol), alkylated (iodoacetamide), and digested (trypsin) to generate peptides, then desalted (SepPak tC18 cartridges) and lyophilised to dryness. For TMT® labeling, peptides in each sample were re-suspended in KH2PO4 buffer, mixed with TMT® 11plex reagents (1 tag per sample according to the labeling plan) and incubated for 1 h at RT. The TMT® reactions were stopped by adding hydroxylamine, and the samples were pooled according to the labeling plan to generate three TMT® 11plex samples which were purified by solid-phase extraction.

Each of the three purified TMT® 11plex samples (~ 250 µg per sample) was fractionated by HPLC-assisted basic reversed phase (bRP) chromatography (EC 250/4.6 Nucleodur C18 Gravity (Macherey-Nagel) and HPLC system (Waters Alliance 2695). In total, 54 tubes were collected at regular time points along the main elution profile for the separation. These were combined to generate 30 fractions per TMT® 11plex sample. Fractions were lyophilised to completion and stored at -80 °C prior to mass spectrometry.

Each of the 30 fractions generated per TMT® 11plex sample was analysed by LC-MS/MS using the EASY-nLC-1000 system coupled to an Orbitrap FusionTM TribridTM Mass Spectrometer (both Thermo Scientific). Re-suspended peptides were loaded onto a nanoViper C18 Acclaim PepMap 100 pre- column (Thermo Scientific) and resolved using an increasing gradient of ACN in 0.1% Formic acid through a 50 cm PepMap RSLC analytical column (Thermo Scientific) at a flow rate of 200 nL/min. Peptide mass spectra were acquired throughout the entire chromatographic run (120 min), using a top speed higher collision induced dissociation (HCD) method Fourier-transform mass spectrometry (FTMS). MS2 scans were acquired at 30,000 resolving power at 400 m/z, following each FTMS scan (120,000 resolving power at 400 m/z).

### MS quality controls

As reported, Proteome Sciences (UK) workflow includes several quality controls of the MS procedures as follows:

Basic Reverse Phase (bRP) Fractionation: chromatograms of the three TMT® 11plex experiment samples were consistent and passed internal quality assessment. Fraction collection (30 fractions per plex) was successfully conducted and 90 fractions were generated from all the three TMT® 11plexes.

TMT®MS2 Analytical QC: Analysis of TMT® labeling reaction efficiency of the three TMT® 11plex experiment samples showed that > 98% of N-terminal amino groups were labelled which indicates that labeling was essentially complete. All MS runs of the fractionated samples passed internal quality assessments based on the total ion counts (TICs) and numbers of peptide spectral matches (PSMs) (data not shown).

MS instrument performance quality control: Quality controls (commercial digest of bovine serum albumin (BSA)) were run before and after samples to check the analytical reproducibility of the MS performance. Retention time stability, intensity values extracted from six monitored BSA peptides, the numbers of peptides and PSMs obtained by HCD fragmentation were within quality requirements (data not shown).

### Computational Mass Spectrometry

In total, 90 separate raw mass spectrometry data files (30 fractions per TMTplex) were submitted to Proteome Discoverer (PD) v2.1 (Thermo Scientific) using the Spectrum Files node. The Spectrum Selector was set to its default values while the SEQUEST HT node was suitably set up to search data against the human FASTA UniProtKB/Swiss-Prot database (version October 2018). The reporter ions quantifier node was set up to measure the raw intensity values for TMT® 11plex mono-isotopic ions (126, 127 N, 127 C, 128 N, 128 C, 129 N, 129 C, 130 N, 130 C, 131, 131 C). The SEQUEST HT search engine was programmed to search for tryptic peptides (with two missed cleavages) and with static modifications of carbamidomethyl (C), TMT6plex (K), and TMT6plex (N-Term). Dynamic modifications were set to deamidation (N/Q), oxidation (M). Precursor mass tolerance was set to 20ppm and fragment (b and y ions) mass tolerance to 0.02Da. All raw intensity values were exported to tab delimited text files for later processing and filtering. Grouped protein results were exported to tab-delimited “Multi-consensus.txt files”, filtered at 1% (High confidence) false discovery rate (peptide spectral matches -PSM- level) and 1 x Rank 1 peptide per protein. Protein grouping was performed using the Parsimony Principle option in the Protein Grouping area within PD. More information about the protein grouping algorithm can be found in the Proteome Discoverer (PD) Version 2.1 User Guide (version A, July 2015).

The steps of data assembly were:

(i) Only none redundant PSMs with protein accession number annotation were used for quantification, (ii) Filtering of PSMs was conducted using Isolation interference information from input Proteome Discoverer multi-consensus file. The threshold of 45% was selected based on analysis of the Isolation interference density distribution. (iii) Isotope impurity correction was applied to PSM level data. (iv) Intensities of the reporter ions of each sample were median-scaled. Then, ratios of reporter ion intensities were calculated for experimental samples relative to the reference sample and log2-transformed. (v) Data belonging to identical peptide sequences were summarized by the median of PSM ratio values to transform the PSM data matrix into a peptide matrix. (vi) The Laboratory Information Management Systems (LIMS) entries were then combined with this peptide matrix to generate a table of peptide identification information (including assigned protein group), quantitative peptide data (given as median PSM ratios in log2 range) together with the sample IDs. (vii) Final peptide data matrix was of size 54,770 peptides and 30 samples.

### MS Data Pre-processing

The following data-dependent pre-processing steps were applied:

(i) Peptides with more than ~ 36% missing values per treatment group were removed, resulting in a reduced peptide data matrix of 30,263 peptides and 30 samples. Remaining missing values were imputed by iterative principal component analysis (iPCA) [31].
(ii) Peptide ratios were quantile normalized.
(iii) Exploratory analysis of the resulting data set revealed that the strongest factor driving non-biological variance within the data is TMT plex effect. Besides this, a minor effect of Medical Centre (Toledo vs. Murnau) was detected. Therefore, batch correction for TMT plex and medical centre effects was applied using LIMMA R package [32]. Exploratory analysis after batch effect removal showed that the clustering of the samples was majorly driven by the clinical outcome.
(iv) Peptides belonging to non-unique protein groups were filtered out, resulting in peptide matrix of size 27,311 peptides (30 samples). The peptide matrix was further used for biological functions enrichment analysis.
(v) To obtain a quantitative protein data matrix, the peptide values from unique protein group peptides were summarized by trimmed mean into a protein value. The protein data matrix of size 2649 proteins (30 samples) was further used for statistical analysis.

Data Quality Control: Quality parameters were controlled during the whole data pre-processing workflow. Data matrices were also analysed using principal component analysis (PCA) with the aim to identify outlier samples. No outlier samples were detected.

### Differential expression statistical analysis

Linear models were created using LIMMA R package [32] to find out peptides and proteins related to neurological recovery (NR vs. SR, as explained before). Models included neurological recovery, level of injury (tetraplegia vs. paraplegia), medical centre (Murnau or Toledo), patient’s sex and age, the time the patient’s sample was stored frozen and, in the case of proteins, also patient’s AIS grade at 30 dpi (A or B). Log_2_ fold change (logFC) and modified t-statistics of NR vs. SR were calculated using LIMMA R package based on the generated linear models.

Setting of log fold change thresholds (FCT) for peptides or proteins were based on the distribution of the standard deviations of every peptide/protein across the 30 patients. Thresholds were adjusted to the median variance level within the data as,

1. log_2_(FCT(peptide/protein)) = 1.47 x median SD (peptide/protein).

As a result, a FCT = 1.8 for peptides and FCT = 1.6 for proteins were applied to the data analysis. Similarly, a p-value threshold of 0.01 was established for peptides and 0.05 for proteins. Multiple testing corrections were performed using the Benjamini-Hochberg procedure.

### Statistical power analysis

One of the most prominent *limma* package features is that inference is reliable even in experiments with small sample size due to the use of empirical Bayes posterior variance estimation. Nevertheless, we performed *a posteriori* estimation of the statistical power achieved for protein-level analysis based on the method for calculating sample size while controlling false discovery rate developed by Liu et al. [33] and implemented into the *ssize.fdr* R package [34]. For this estimation, the effect size was set to 0.68 (log_2_ FCT, Eq. (1)), FDR to 0.1 (value used to filter differentially expressed proteins, Table 2) and statistical test was determined as two-sided. The standard deviation (SD) was fixed to the median SD of all 2649 proteins across the 30 patients (0.4375), and π_0_ (proportion of null p-values) was estimated from *limma* analysis as 0.66 using the *qvalue* R package [35]. Based on these parameters, the statistical power achieved by including 10 patients –sample size of the smallest group: strong recovery– is 0.91, quite above the standard value of 0.8 commonly used in sample size calculation. Indeed, based on the same parameters, a statistical power of 0.8 (actually 0.81) is expected to be reached by including 8 patients in each experimental group.

**Table 2:** Blood serum low abundant proteins differentially enriched in strong recoverers (SR) or non-recoverers (NR)

Gene name	Protein name	log_2_FC (NR vs. SR)	SR enrichement (folds)	NR enrichment (folds)		*p*-value	FDR	ELISA
CALU	Calumenin	-1.047	2.07			2.6E-05	0.037	✓*
SERPINE1	Serpin family E member 1	-1.144	2.21			2.8E-05	0.037	✓
CTSW	Cathepsin W	-0.735	1.66			1.6E-04	0.068	
RAP1B/RAP1A	Ras-related protein Rap-1B	-0.700	1.62			2.3E-04	0.068	
ANGPT1	Angiopoietin-1	-0.748	1.68			2.4E-04	0.068	✓
ARHGAP35	Rho GTPase activating protein 35	-1.587	3			6.8E-04	0.091	✓
FKBP4	FKBP prolyl isomerase 4	-0.945	1.92			7.4E-04	0.091	
FHOD1	FH1/FH2 domain-containing protein 1	-0.794	1.73			7.5E-04	0.091	
B4GALT7	Beta-1.4-galactosyltransferase 7	-0.979	1.97			8.7E-04	0.094	
CNDP1	Beta-Ala-His dipeptidase	-0.713	1.64			9.9E-04	0.094	
PIN1	PDZ domain containing 2	-1.158	2.23			1.2E-03	0.094	✓
TCN2	Transcobalamin-2	-0.700	1.62			1.6E-03	0.096	✓
AGER	Advanced glycosylation end product-specific receptor	0.968			1.96	5.4E-05	0.048	✓
DEFA1/DEFA3	Defensin alpha 1 / Defensin alpha 3	1.147			2.21	4.1E-04	0.078	✓
ITPA	Inosine triphosphate pyrophosphatase	0.826			1.77	6.5E-04	0.091	
BPIFB2	BPI fold containing family B member 2	1.419			2.67	1.0E-03	0.094	
UPB1	Beta-ureidopropionase	0.806			1.75	1.2E-03	0.094	
ADM	Pro-adrenomedullin	0.689			1.61	1.3E-03	0.094	
RACK1	Small ribosomal subunit protein RACK1[…]	1.067			2.09	1.4E-03	0.095	✓*
STXBP5	Syntaxin binding protein 5	0.897			1.86	1.4E-03	0.095	
LSM1	LSM1 homolog. mRNA degradation associated	0.922			1.89	1.5E-03	0.095	
OLR1	Oxidized low-density lipoprotein receptor 1	1.125			2.18	1.7E-03	0.096	✓*
CTSG	Cathepsin G	0.973			1.96	1.7E-03	0.096	✓
CD300A	CMRF35-like molecule 8	0.900			1.86	1.7E-03	0.096	✓

✓ Selected for ELISA validation; * Current available kits not sensitive enough for a reliable evaluation of the protein in our samples

### Functional analysis

Functional analysis was performed at the peptide level to identify biological processes that are significantly altered between the different samples, where the applied set of thresholds was the same as during statistical testing: For peptides *p* < 0.01; |FC|>1.8)

A Significance of Enrichment analysis, based on the Fisher Exact Test, was performed by means of a tool developed by Proteome Sciences (Functional Analysis Tool; FAT v1.2.0). Enrichment of functional terms: Gene Ontology Biological Processes and Biological Pathways was performed within FAT. A two-sided p-value was generated by the Fisher’s exact test and the Benjamini-Hochberg method was used for multiple test correction. A minimum of two matched identifiers (e.g. gene names) was required and terms with an adjusted significance value < 0.3 were considered significant. All functional results were visualized using volcano plots (enrichment vs. adjusted p-value).

Gene Ontology (GO) term and pathway enrichment were performed using the background of all non-regulated peptides identified in the study. FAT calculates an enrichment or depletion of annotation terms among the regulated peptides/proteins, where “regulated” implies those passing the fold change thresholds, as broadly used in gene set analysis [36].

### Validation of selected proteins by ELISA

We used specific ELISA kits to validate proteins that were enriched in patients with strong recovery or with no recovery constrained to a false discovery rate (FDR) < 0.1. The following kits were used following manufacturer instructions (the dilution of our sera used for each kit is also detailed): AGER (R&D Systems, R&D Systems, Minneapolis, MN, USA; #DRG00; 1:1 dilution), ANGPT1 (RayBiotech, Peachtree Corners, GA, USA; #ELH-Angiopoietin1-1; 1:35 dilution), ARHGAP35 (Abbexa, Cambridge, UK; #abx384958; 1:15 dilution), CALU (Aviva Systems Biology, San Diego, CA, USA; #OKEH04727), CD300A (RayBiotech, Peachtree Corners, GA, USA; # ELH-CD300A-1; 1:10 dilution), CTSG (Aviva Systems Biology, San Diego, CA, USA; #OKEH01241; 1:750 dilution), DEFA1/DEFA3 (Aviva Systems Biology, San Diego, CA, USA; #OKBB01048; 1:500 dilution for SCI and SPFC groups, 1:300 for HC), OLR1/ LOX-1 (RayBiotech, Peachtree Corners, GA, USA; #ELH-LOX1-1*);* PIN1 (Aviva Systems Biology, San Diego, CA, USA; #OKCD06255; 1:100 dilution), RACK1/ GNB2L1 (RayBiotech, Peachtree Corners, GA, USA; #ELH-GNB2L1-1; 1:6), SERPINE1/PAI1 (Aviva Systems Biology, San Diego, CA, USA; #OKCD06428; 1:100 dilution), TCN2 (Aviva Systems Biology, San Diego, CA, USA; #OKEH02273; 1:500 dilution). Plates were read at 450 nm in a Spark® Multimode Microplate Reader (Tecan Austria GmbH, Grödig, Austria). The dilution of sera stated above for every ELISA determination was determined after testing a range of dilutions (up to eight) for each kit, to ensure that the analyte concentration was within the range of the standard curve.

Standard curves were performed in every plate according to the manufacturer instructions and optical densities were adjusted to the most appropriate model (that with the highest coefficient of determination, r^2^) for interpolating the analyte concentration. For all cases but CTSG and SERPINE1, a 5-parameter logistic regression curve was fitted to the standard curve. For CTSG and SERPINE1, a simple linear regression was fitted. In all cases, r^2^ > 0.99.

All samples were measured by duplicate and technical reproducibility (inter and intra-assay) was checked by measuring the coefficient of variation (CV; mean value across all samples and ELISAs 8.5%).

A limit of detection (LoD) was established independently for every plate as suggested by the guidelines of the Clinical and Laboratory Standards Institute [37]. First a limit of blank (LoB) was established as:

(2) LoB = Mean_blank_ + 1.645 SD_blank_.

Then,

(3) LoD = LoB + 1.645 SD _lowest concentration standard_.

Whenever a sample was under the LoD, values were dismissed and new ELISAs were performed concentrating these samples.

Levels of analytes measured by ELISA were tested for normality by Shapiro’s test. Whenever normality could not be assumed, levels of analytes between NR and SR were compared by Mann-Whitney’s test; otherwise, Student’s t-test was applied. The same procedure was applied to compare levels of patients with those of control subjects. The precise statistical test is stated in the correspondent figure legend. All statistical analysis were performed in R statistical programming language [38] using RStudio [39].

## Results

As a first step, blood samples drawn 4 weeks after injury from patients with no clinical recovery (*n* = 20) and with strong recovery (*n* = 10) were analyzed via the presented untargeted approach.

**1. Peptidomics identifies inflammation, lipid metabolism and coagulation as main processes related to neurological recovery after SCI.**

**Fig. 2:**
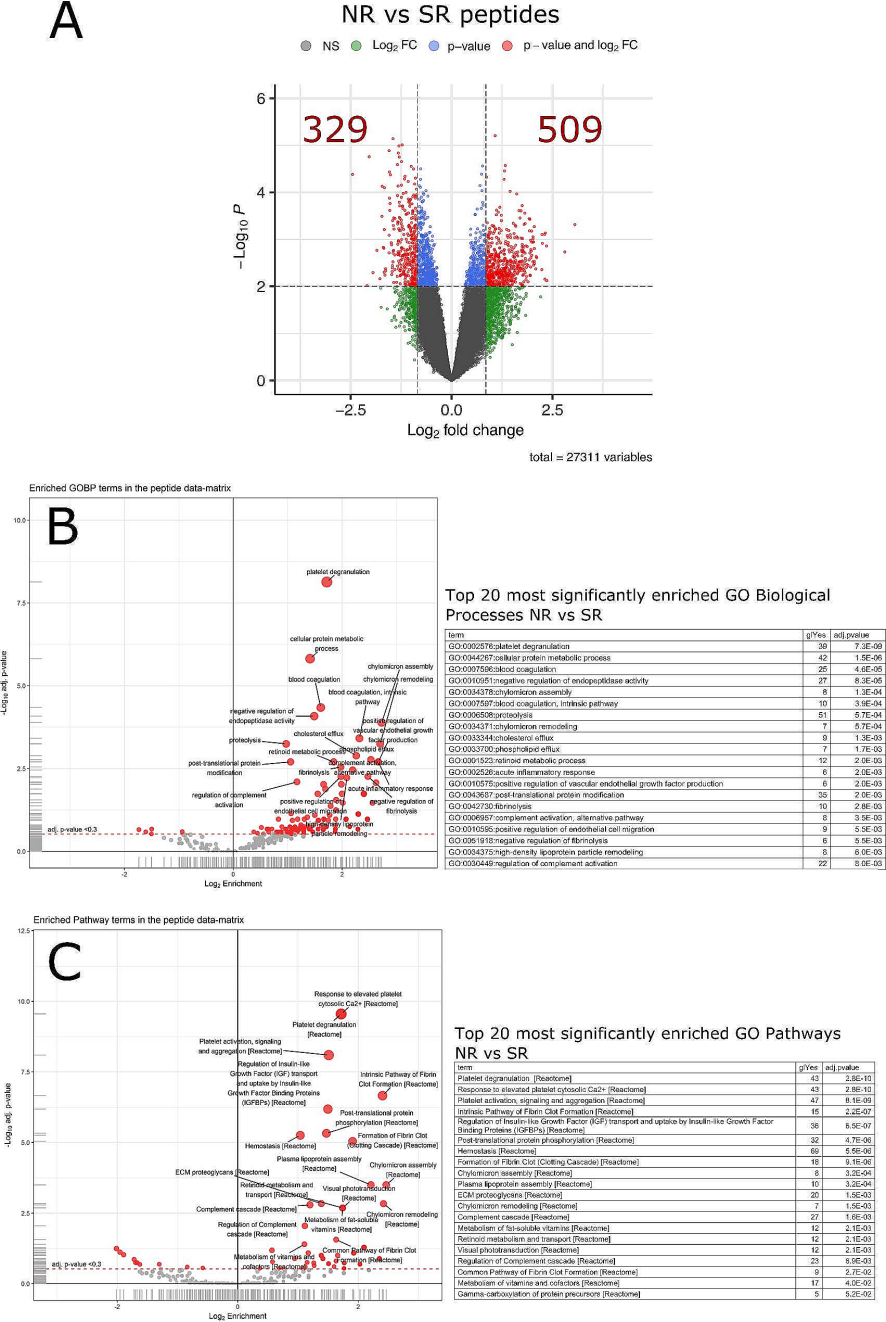
Analysis of peptides with differential expression between patients with no (NR) or strong (SR) recovery. **(A)** Volcano plot for data comparisons at peptide level. The plot shows features log_2_ FC values (x-axis) and log_10_ p-values (y-axis). The thresholds applied (dashed lines) are alpha = 0.01 and FCT = 1.8. Spots in red indicate peptides that fulfil the p-value and FC threshold criteria (quantity shown in red numbers). Blue spots highlight significant peptides (p-value < = alpha) that fall below the FC threshold. Green spots represent peptides with statistical significance but below FC threshold criteria and grey spots those that did not meet any significance criteria. **(B, C)** Functional analysis using enriched peptides. Volcano plots and extracts of the enriched tables based on regulated peptides are shown for **(B)** Gene Ontology Biological Processes (GO_BPs) and **(C)** Pathways. For each analysis, red filled circles indicate terms found to be over-represented in the data set. Tables with top 20 enriched terms are reported for each comparison where the terms are sorted by the adj-p-values

Analysis of the 30 human HAP-depleted serum samples resulted in the detection of 54,770 unique peptide sequences representing 4,800 unique protein groups. Of these, 27,311 peptides (2,649 protein groups) were finally quantified and statistically evaluated in all 30 patients. Statistical analysis showed 838 peptides with significant fold-changes (> 1.8-fold) and p-value (< 0.01) when comparing strong recovery (SR) vs. no recovery (NR; Fig. 2A), among which 329 were enriched in SR and 509 in NR (Fig. 2A).

Functional enrichment analysis of these 838 peptides were performed for gene ontology biological processes (Fig. 2B) and pathways (Fig. 2C). Results are based on regulated peptides and presented as volcano plots and extracts of the enriched tables where the terms are sorted by the adjusted p-values.

Patients with NR show significant enrichment vs. those with SR in (i) biological processes related with coagulation and platelet function *(enriched terms: “platelet degranulation”, “blood coagulation”, “blood coagulation intrinsic pathway”, “fibrinolysis”, “negative regulation of fibrinolysis”*), (ii) lipid metabolism *(“cellular protein metabolic process”, “negative regulation of endopeptidase activity” and “chylomicron assembly”, “chylomicron remodeling”, “cholesterol efflux”, “high density lipoprotein remodeling*”), and (iii) inflammation (*“acute inflammatory responses”, “complement activation, alternative pathway”, “regulation of complement activation”)* (Fig. 2B). Similar results were obtained with pathways analysis: NR show significant over-representation in (i) platelet aggregation and clot formation *(“platelet degranulation”, “Response to elevated platelet cytosolic Ca2+”, “Platelet activation signaling and aggregation”, “Intrinsic Pathway of Fibrin Clot Formation”, “Hemostasis”, “Formation of Fibrin Clot”, “Common Pathway of Fibrin Clot Formation”*), (ii) lipoprotein formation (“*Chylomicron assembly”, “Plasma lipoprotein assembly”, “Chylomicron remodeling”*), (iii) metabolism of liposoluble proteins (*“Metabolism of fat-soluble vitamins”, “Retinoid metabolism and transport, “Metabolism of vitamins and cofactors”*), (iv) inflammation, specifically related to the Complement system *(“Complement cascade”, “Regulation of Complement cascade”*), extracellular matrix (“*Extracelllular matrix Proteoglicans*”) and (v) IGF/Insulin metabolism (*“Regulation of Insulin-like Growth Factor (IGF) transport and uptake by Insulin-like Growth Factor Binding Proteins (IGFBPs)”*).

Since an association between the occurrence of Deep Vein Thrombosis (DVT) and the levels of some coagulation factors after SCI has already been described [40], we checked the record of DVT events previous to serum sampling in our cohort. We found only one patient with a DVT record before 30 days. We also looked for DVT events after serum sampling, during the time interval used for classifying the neurological recovery of patients (30–120 days after SCI) and found non-significant differences between NR and SR (Fisher’s exact test *p* = 0.24).

For a better interpretation of the data, we also wanted to rule out possible differences in the number of NR or SR patients receiving anti-coagulant therapy at the time of serum sampling. Most patients in both groups (more than 70% of patients) were using low molecular heparin analogues (factor Xa inhibitors). No significant differences were found between the groups (Fisher’s exact test *p* = 0.77).

**2. Differential expression at proteomics level also shows significant enrichment of inflammation and coagulation related-proteins between SR and NR.**

**Fig. 3:**
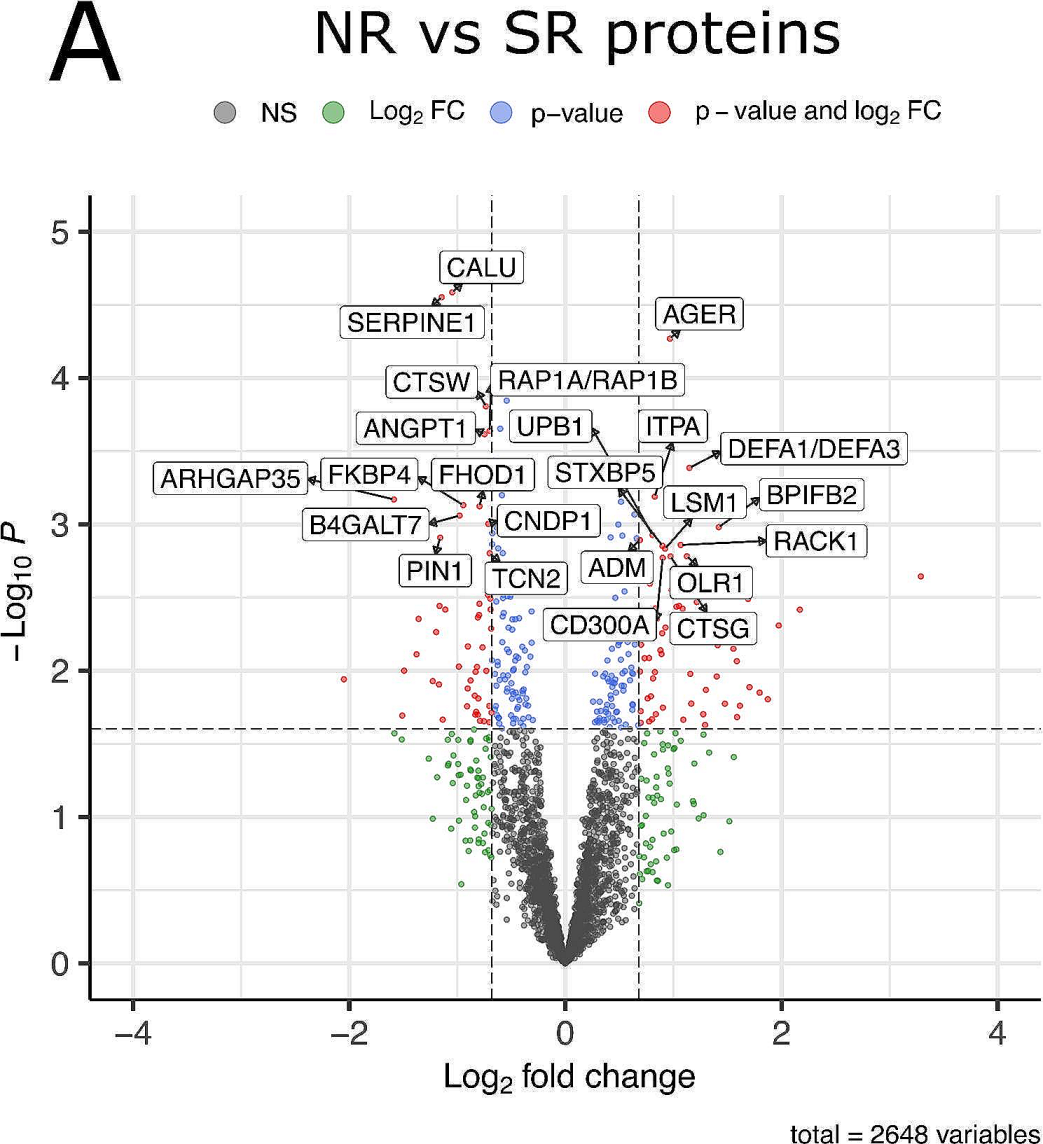
Analysis of the proteins enriched in NR (positive values in x-axis) or SR (negative values in x-axis). Only the name of proteins with FDR < 0.1 are shown. The thresholds applied (dashed lines) are alpha = 0.05 and FCT = 1.6. Spots in red indicate proteins that fulfil the p-value and FC threshold criteria; blue spots highlight significant peptides (p-value < = alpha) that fall below the FC threshold; green spots represent peptides with statistical significance but below FC threshold criteria and grey spots, those that did not meet any significance criteria

When searching for new biomarkers and therapeutic targets, peptidomics results should be translated to the protein level. In this way, we identified 154 proteins with significant fold-changes (> 1.6-fold, i.e. logFC > 0.68) and p-values (< 0.05; Fig. 3) (Supplementary Table S3). Of them, 76 were enriched in SR and 68 in NR (Fig. 3). Further filtering by a false discovery rate (FDR) < 0.1 results in a set of 24 proteins (Fig. 3; Table 2). Among these, three had a FDR < 0.05: calumenin (CALU), SERPINE1 and AGER (RAGE). The first two proteins are enriched in SR and the third one, in NR. In addition, CALU shows the highest logFC ratio among the 24 proteins (logFC = 2.25 vs. SR, meaning 4.75-fold higher levels than NR). About the remaining 21 proteins, (Table 2), 10 are enriched in SR (ANGPT1, ARHGAP35, B4GALT7, CNDP1, CTSW, FHOD1, FKBP4, PIN1, RAP1B/RAP1A, TCN2) and 11 in NR (ADM, BPIFB2, CD300A, CTSG, DEFA1/DEFA3, ITPA, LSM1, OLR1, RACK1, STXBP5, UPB1). Among them, ARHGAP35 and BPIFB2 stand out by their logFC ratio (ARHGAP35 logFC = 1.58, meaning 2.99-fold increase vs. NR; BPIFB2 logFC = 1.42, meaning 2.67-fold increase vs. SR).

Of the significantly enriched proteins with a FDR > 0.1, immunoglobulin heavy and light chains are enriched in NR between 1.8 and 3.5 fold (IGHA2, logFC = 1.80; IGHV41, logFC = 1.56; IGLV4, logFC = 1.59; IGKV21, logFC = 1.27; IGLV11, logFC = 1.09; IGHV11, logFC = 1; IGLV22, logFC = 0.82). The complete list of significantly enriched proteins can be found in Supplementary material (Supplementary Table S3).

**3. Technical validation of proteomics by ELISA.**

**Fig. 4:**
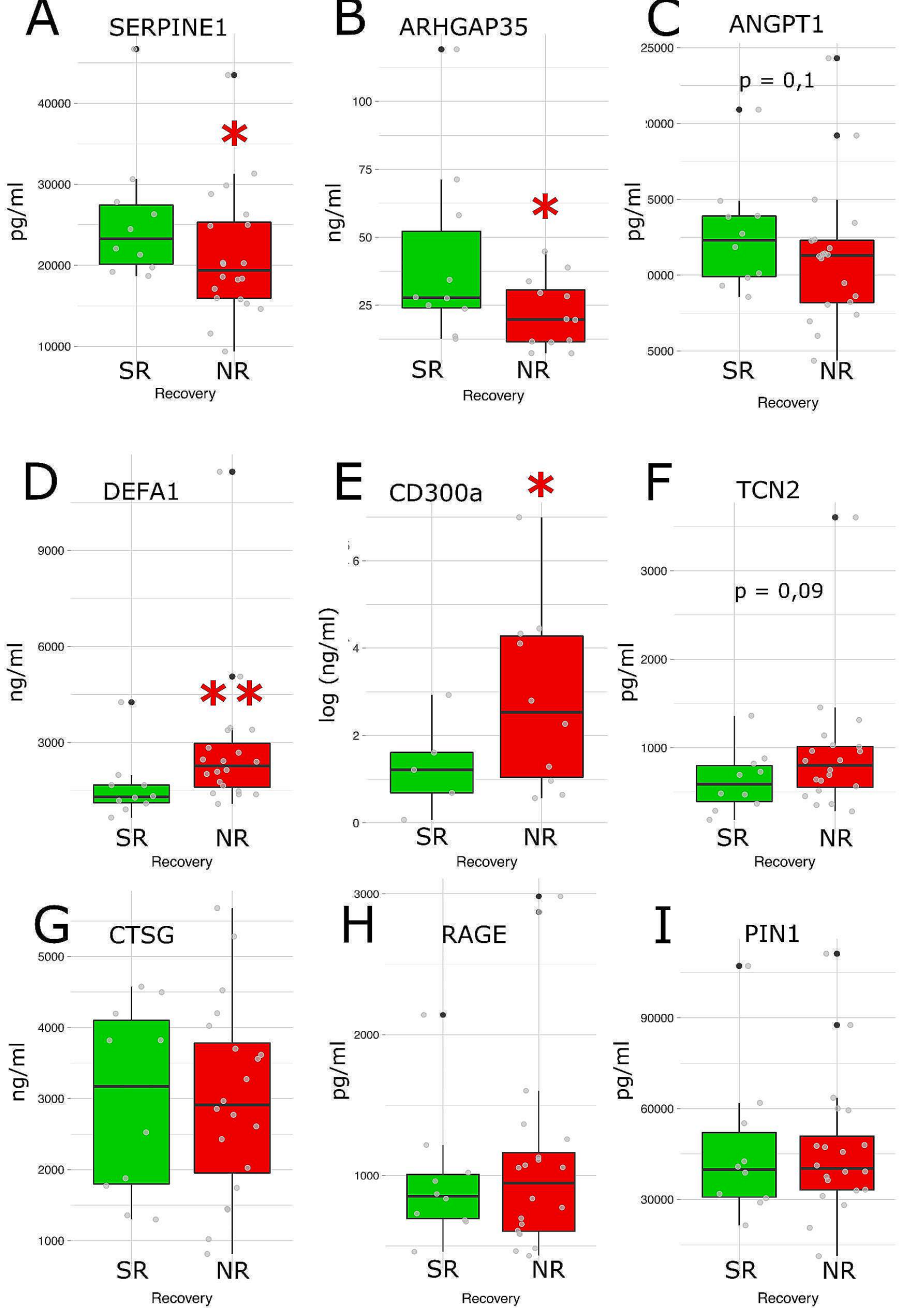
Enzyme-linked immunosorbent assays (ELISA) validation and quantification of serum levels of proteins differentially expressed between NR (red) and SR (green). Statistical analysis corroborated the presence of significant higher levels in SR of SERPINE1 **(A)** and ARHGAP35 **(B)** and significant higher levels of DEFA1 **(D)** and CD300a **(E)** in NR. Mann-Whitney test was conducted in **A, B, C, D, F, H** and **I**. Student’s t-test was applied in E and G. * *p* < 0.05 vs. SR; ** *p* < 0.01 vs. SR. **ADDITIONAL MATERIAL**: **Additional File 1**: File name: SUPFIG1 MOD.tif File format: .tif

In order to validate the result of our untargeted approach with additional techniques and to quantify the levels of enriched proteins, we selected a subset of them among those with a FDR < 0.1 to quantify their serum levels by ELISA. We chose proteins with at least 2-fold difference between NR and SR groups (Table 2). Due to the limited amount of serum available and the volume requirements for each kit, we restricted the analysis to a maximum of 11 proteins: six increased in NR (DEFA1/3, OLR1, RACK1, CTSG, AGER, CD300A) and five increased in SR (CALU1/CALU, ARHGAP35, PIN1, SERPINE1, TCN2). Unfortunately, the sensitivity of the current available kits for calumenin (CALU), Small ribosomal subunit protein RACK1 (RACK1) and Oxidized low-density lipoprotein receptor 1 (OLR1) (asterisk in Table 2) were not sufficient to reliably evaluate the levels of those proteins in sera. Among the other tested proteins, four reached statistical significance: two enriched in SR (SERPINE1 and ARHGAP35; Fig. 4) and two enriched in NR (DEFA1/3 and CD300a; Fig. 4).

**4. Comparison of the levels of proteins differentially enriched in NR or SR versus those of control subjects.**

The levels of proteins significantly different between NR and SR by ELISA were compared with those of healthy controls (HC) and patients with spinal fracture but without neurological deficits (SPFC) (Table 3). No difference in the levels of these proteins (ARHGAP35, CD300, SERPINE1, DEFA1) were found between HC and SPFC. However, all the protein levels in NR, except ARHGAP35, were significantly higher than those of both HC and SPFC (CD300, SERPINE1 and DEFA1), suggesting a neurogenic-driven increase of all of them three. On the other hand, only SERPINE1 levels were significantly higher than those of HC and SPFC in SR, whereas CD300a and DEFA1 show significance only vs. HC. ARHGAP35 levels in SR were not different from control levels.

**Table 3:** Serum levels of proteins confirmed by ELISA to be related to neurological recovery of patients (no recovery (NR) vs. strong recovery (SR)), compared with those in healthy controls (HC) and patients with spine fracture without neurological damage (SPFC).

	HC	SPFC	SCI NR	SCI SR
TCN2 (pg/ml)	532 ± 27	525 ± 65	921 ± 158 ** ^	627 ± 109
ARHGAP35 (ng/ml)	25.2 ± 3.1	29.1 ± 7.1	22 ± 3.7	41.3 ± 10.4
CD300a (ng/ml)	1.27 ± 0.18	1.66 ± 0.65	14.93 ± 8.89 ** ^	3.13 ± 1.17 *
SERPINE1 (ng/ml)	13.6 ± 0.6	14.3 ± 1.4	21.2 ± 1.7 *** ^	25.7 ± 2.6 *** ^^^
DEFA1 (ng/ml)	934 ± 64	1238 ± 273	2797 ± 503 *** ^^	1602 ± 319 **

Mann-Whitney’s test significance: * *p* < 0.05 vs. HC; ** *p* < 0.01 vs. HC; *** *p* < 0.001 vs. HC; ^ *p* < 0.05 vs. Spine Fracture; ^^ *p* < 0.01 vs. Spine Fracture; ^^^ *p* < 0.001 vs. Spine Fracture

HC: healthy controls; SPFC: patients with spine fracture without neurological damage; NR: SCI patients with no recovery; SR: SCI patients with strong recovery

## Discussion

In the complex field of spinal cord injury there is an increasing interest in finding biomarkers that may help to evaluate the clinical severity of the lesion in a specific patient, to foresee the recovery expectancy for a realistic rehabilitation strategy and to unravel pathophysiological mechanisms of pathology, indicating new therapeutic targets. The search for new blood biomarkers in SCI is hampered, however, by the overwhelming presence of high and medium abundant proteins (HAPs and MAPs) that greatly masks others with much lower, but relevant, representation (low abundant proteins, LAPs). Here we used a tandem depletion strategy that uncovered new LAPs differentially present in the blood of patients with no spontaneous recovery versus those with strong recovery in the subacute phase (from 30 to 120 days after injury). The strategy we used for depletion may hold intrinsic limitations, like the loss of relevant proteins that may be associated to those depleted [41], but it was still needed to detect many others, as shown. Some of these proteins were further validated here using complementary techniques, like ELISA, making it easier to implement for a regular use across laboratories and clinical settings.

Three proteins show high significant differential expression between groups: calumenin (CALU), SERPINE1 and RAGE. CALU and SERPINE1 are enriched in SR, whereas RAGE is higher in NR, although was not further validated by ELISA (Fig. 4).

**CALU** shows the highest ratio between SR and NR among all the proteins (4.75-fold higher in SR). Calumenin is a protein that belongs to the CREC family of low-affinity Ca(2+)-binding proteins, and is secreted by mammalian cells [42, 43]. CALU is found in stimulated platelets of rats and humans, and opposes the anticoagulant action of warfarin by inhibiting its interaction with the vitamin K epoxide reductase [44]. It can also act in the liver, where it is closely associated with inflammatory states or cirrhosis [45]. Calumenin may also interact with serum amyloid P component (SAP), a glycoprotein mainly synthesized in the liver, and may participate in the immunological defense system and be involved in the pathological process of amyloidosis that leads to formation of amyloid deposits seen in different types of tissues [46]. Unfortunately, the sensitivity of the current available CALU tests did not allow us to validate and find the absolute levels in the different groups by ELISA, neither establish the relationship with control individuals, but it seems an outstanding candidate that warrants future insight.

**SERPINE1, Serpin Family E Member 1**, also known as PAI-1 (Plasminogen Activator Inhibitor 1), is a member of the serine protease inhibitor (serpin) superfamily. SERPINE1 is the principal inhibitor of the plasminogen activators that catalyze the activation of the potent fibrinolytic factor plasmin. Therefore, changes in the levels of SERPINE1 affect the hemostasis and lead to bleeding or thrombotic complications [47, 48]. We found that SERPINE1 levels are significantly higher in SCI patients versus controls (healthy and spine fracture without neurological impairment), and, among SCI patients, SERPINE1 is higher in SR vs. NR. This was initially surprising for us, since both GO terms and the levels of other specific proteins suggest that NR show a pro-coagulative profile compared to SR. NR peptidomics is enriched in GO terms like *“platelet activation”*, “*aggregation and degranulation”, “blood coagulation”, “formation of fibrin clot formation”* and *“inhibition of fibrinolysis”.* Why may SERPINE1, a classical anti-fibrinolytic agent, be higher in SR, then?

Platelet function and coagulation are dynamic and complex processes with a high relevance after trauma and, specifically, SCI. In these pathologies, two apparently opposed states appear that have to be faced clinically, and may even occur simultaneously: hypocoagulopathy, that may lead to hemorrhage and serious complications; and hypercoagulopathy, that may lead to thrombosis and formation of deep venous thrombosis (DVT) [49]. In traumatic brain injury, management focuses primarily on hypocoagulopathy with prolonged bleeding, although also attends the possible hypercoagulation states that may induce thrombosis [49]. In SCI, however, the high risk of developing venous thromboembolism in the acute phase, that is life-threatening [40, 50, 51], overcomes other considerations and leads to the general recommendation of establishing an anticoagulant prophylaxis within 72 h that may last beyond 6–8 weeks [50, 52]. The causes of high incidence of DVT in acute SCI have been related with the presence of factors characterizing the “Virchow triad” [40]: (i) the stasis of circulating blood due to the loss of muscle pump, dilatation of the blood vessels, and decreased blood flow from the lower limbs; (ii) the presence of hemostasis and higher levels of pro-thrombotic factors in SCI patients (Factor VIII, fibrinogen, levels and aggregation of platelets…); and (iii) the damage to venous endothelial cells [40]. However, important adaptations occur in SCI patients over time that turns VTE risk in the chronic stage similar to that of the general population, despite long-term immobilization [53–56]. Some reasons have been proposed for this: lower levels of pro-coagulation factors, like factor VIII activity, prothrombin fragments 1 + 2 and D-dimer (but not SERPINE1), the loss of normal circadian rhythms that increase coagulation and lower fibrinolysis in the mornings (except for SERPINE1, again, that maintains the morning peak), and the presence of lower HSP47 expression in platelets that prevents interaction with neutrophils and lowers inflammatory prothrombotic events [53, 57]. Previous evidence, therefore, show that SERPINE1 behaves differently to the rest of coagulation/fibrinolytic factors in some aspects and this may be the case also in our patients. Maybe SERPINE1 is accomplishing functions beyond coagulation, as described [47, 48], and may be related to conditions such as cytokine hyperproduction, infections or metabolic syndrome [47, 48]. Or maybe, the exact equilibrium between coagulation and fibrinolysis, between helping damage repair and hemostasis vs. prevention of clot formation, requires complex coordination of factors, in which elevated SERPINE1 is needed and this is related to a better general outcome in patients. It must be considered that we measured SERPINE1 during the subacute stage, i.e. in the midway between the coagulatory state derived from the acute insult and the adaptations to the chronic SCI stage. At this time, also, all our patients still received anticoagulatory treatment and maybe the overproduction of SERPINE1 is a reactive response to this pharmacologically-induced state, and higher levels in SR provides them a better compensation thereby resulting in a better sensorimotor performance. The relation between platelet function, coagulation and neurological recovery in SCI is still largely unknown. In past reports, SERPINE1 levels are reduced after exercise training in chronic SCI in parallel to obesity-related markers, but this reduction has not been related to neurological function [58]. There is evidence that a lower number of platelets in the sub-acute stage of SCI correlates with better ability to walk [2], but the contribution of each coagulation factor to functional improvement, including SERPINE1 warrants further research. It must be said that we provide evidence here based on sera, therefore missing a number of factors that remain in the blood clot during the sample processing. New studies using also plasma and covering the whole repertoire of pro and anti-coagulant factors are needed to definitely unravel the link between coagulation/fibrinolysis and recovery.

**CD300a** belongs to the CD300 family of molecules, in the immunoglobulin superfamily (IgSF) receptors [59, 60]. CD300 family consists of eight members, among which CD300a and CD300f are functional orthologs, with intracellular inhibitory signaling domains. CD300a play important roles in regulating activation, proliferation, differentiation, migration and immunity function of leukocytes, mainly among the myeloid lineage [59–61]. CD300a recognizes amino phospholipids, especially phosphatidyl serine and phosphatidyl ethanolamine, which are exposed on the surface of dead and activated cells [62]. Although CD300a has been described as a transmembrane receptor, we detected it in patients’ sera. This could be explained as being part of cell debris due to the injury, although this seems unlikely, since it might have been removed at the time at which we collected the serum (30 days after SCI). The possibility also exists that we are measuring a soluble form of CD300a. Even though soluble CD300a has not been described to date, to the best of our knowledge, soluble forms for other CD300 members, like CD300b have been found recently, being released by neutrophils in response to lipopolysaccharide (LPS) to amplify lethal inflammation in sepsis [63]. This would indicate a similar role as the transmembrane receptor, since CD300b belongs to the activation receptor repertoire. In that regard, the higher levels of CD300a in NR patients (4 fold vs. SR and up to 10 fold vs. controls) could be related to the existence of high levels of PS or PE derived from delayed cell death or axonal degeneration [64] which can be found at this time point [65]. This might be a reactive response, directed to counteract an excessive or inappropriate activation of the myeloid lineage (neutrophils, basophils, eosinophils, monocytes…) in contrast to SR patients, with circulating CD300a levels closer to controls and a lower inflammatory profile. This would be supported by the fact that in other diseases with an important inflammatory component, such as allergy, psoriasis or ulcerative colitis, CD300a indeed decreases the inflammatory state [59]. However, a direct pathological role of high levels of CD300a (like those found in NR) cannot be totally ruled out. For instance, some studies show that CD300a negatively regulates the proliferation of IFN-β-dependent Treg cells, leading to more severe allergic reactions [66]. There is not much evidence to date that can help us to discuss CD300a role after SCI. No studies have focused on circulating levels, and only one describes increases of CD300a after SCI in rodents, without more insight on its functional role [67].

**DEFA1** is a peptide mostly produced by neutrophils with an important microbicidal activity [68]. However is also involved in other processes like liver pathology, where it behaves as anti-steatosis factor but shows an important pro-fibrotic effect [69–71]. Neutrophil released defensin alpha is also involved in thrombosis and inhibits fibrinolytic processes [72] and DEFA1 can be found in Neutrophil extracellular traps [73], a major inducer of inflammatory thrombosis [74, 75]. Again, the higher levels of DEFA1 in NR are in accordance with the above discussed higher inflammatory and pro-coagulatory states in NR vs. SR. Accordingly, the levels of DEFA1 in the blood of SCI patients is higher than in controls, but especially in NR blood (around 2800 ng/ml vs. 1600 ng/ml in SR and 1200 ng/ml in spine fracture controls or 900 ng/ml in healthy controls, Table 3). DEFA1 has been shown in neutrophils and macrophages infiltrating spinal cord parenchyma after lesion [76], but not in blood up to date. Further studies are warranted to define the specific role of DEFA1 either as mediator or just a marker of the inflammatory state.

Among the rest of the proteins that maintain significance when considering a FDR = 10%, ARHGAP35 stands out by its logFC ratio (ARHGAP35 logFC = 1.58, meaning 2.99-fold increase SR vs. NR); and this enrichment was validated by ELISA. Interestingly, ARHGAP35 levels in NR were lower than controls, although not significant, whereas in SR were higher (but also not significant vs. controls). However, this difference renders significant higher levels in SR vs. NR, as reported. **ARHGAP35 is a RhoA-GAP specific protein**, that inhibits RhoA activity [77]. It was one of the first RhoA-specific GAP proteins characterized in platelets, in which it facilitates platelet spreading and clot retraction spreading [77]. This would fit with the abovementioned distinct coagulation profile between SR and NR at this time point after lesion.

Among the limitations of the study, it should be noted that the available amount of sera and the volumes required to conduct ELISAs restricted the validation of the 24 proteins with an FDR < 0.1 (Table 2) to 11 selected proteins. Although the selected proteins were chosen based on differential expression and statistical significance, candidate markers of recovery could have been missed among the 13 proteins not tested. Also, the lack of sensitivity of available ELISA tests to detect some of the selected proteins at their concentration in serum –CALU, RACK1 and OLR1– further limited the validation to a final number of 8 proteins.

Also, future studies should be conducted to test whether our results are generalizable in an independent validation cohort. In this regard, validation tests by ELISA or other common clinical laboratory technique should be conducted including other patient profiles, like incomplete lesions. Moreover, conducting new studies with samples obtained at various time points, including the chronic stage of SCI, would elucidate whether the observed changes between NR and SR are temporary or permanent, whether the identified biomarkers are predictive in the long term and may provide additional insights into the dynamics of recovery. In addition, testing other sample types like cerebrospinal fluid or extracellular vesicles, could provide further insights into the origin of the biomarkers highlighted in the present study. Furthermore, integrating the results obtained here with those derived from other -omics, like genomics and metabolomics, could greatly help to depict the pathogenic mechanisms underlying the differences in neurological recovery of patients with SCI.

## Conclusions

In summary, depletion of high and medium abundant proteins allowed us to find low abundant proteins that are differentially present in serum of patients with SCI with no recovery vs. those with strong recovery in the subacute phase (from 30 to 120 days after injury). Among them, CALU, SERPINE1, CD300a, DEFA1 or ARHGAP35 may be good candidates as new biomarkers for neurological recovery or therapeutic targets to be explored in future studies with larger validation cohorts. The enrichment in some of these proteins has been validated with alternative, more clinical-friendly techniques, such as ELISA, facilitating their transit to extended clinical practice.

Finally, our data highlight coagulation and inflammation states in subacute SCI (30dpi) and platelet-neutrophil interaction as potential mechanisms underlying recovery or absence of recovery, that warrant future insight. There is an intense cross-talk between platelet function and inflammation. Specifically, neutrophils can contribute to thrombosis and coagulation by the intrinsic pathway (one of the GO-terms enriched in NR vs. SR) [74, 78], and platelets may activate neutrophils and the delivery of neutrophil extracellular traps, that induce thrombus formation [53, 79].

### Electronic supplementary material

Below is the link to the electronic supplementary material.

Supplementary Material 1

Supplementary Material 2

Supplementary Material 3

Supplementary Material 4

Supplementary Material 5

## Data Availability

The datasets used and/or analyzed during the current study are available from the corresponding author on reasonable request.

## Abbreviations

AIS: American Spinal Injury Impaiment Scale
bRP: Basic reverse phase chromatography
BSA: Bovine serum albumin
DVT: Deep Vein Thrombosis; ELISA
FAT: Functional Analysis Tool
FCT: Log fold change thresholds
FDR: False discovery rate
FTMS: Fourier-transform mass spectrometry
GO: Gene Ontology
HAPs: High abundant proteins may account
HC: Healthy control group
HCD: Higher collision induced dissociation
IGHA2: Immunoglobulin Heavy Constant Alpha 2
IGHV4: Immunoglobulin heavy variable 4
IGHV11: Immunoglobulin heavy variable 11
IGKV2: Immunoglobulin kappa variable 2
IGLV4: Immunoglobulin lambda variable 4
IGLV11: Immunoglobulin lambda variable 11
IGLV22: Immunoglobulin lambda variable 22
INCS: Integrated Neurological Change Score
iPCA: Iterative principal component analysis
ISNCSCI: International Standards for Neurological Classification of Spinal Cord Injury
LAPs: Lower Abundant Proteins
LC-MS2: Liquid chromatography-mass spectrometry
LPS: Lipopolysaccharide
NR: Patients with no recovery
PAI-1: Plasminogen Activator Inhibitor 1
PE: Phosphatidylethanolamine
PS: Phosphatidylserine
PSMs: Peptide spectral matches
SCI: Spinal cord injury
SPFC: Spine fracture control group
SR: Patients with strong recovery
TMT: Tandem mass tagging
