# Supplementary figures and images for: Untargeted blood serum proteomics identifies novel proteins related to neurological recovery after human spinal cord injury
Source: J Transl Med. 2024 Jul 17;22:666. doi: 10.1186/s12967-024-05344-y (PMC11256486; doi:10.1186/s12967-024-05344-y)

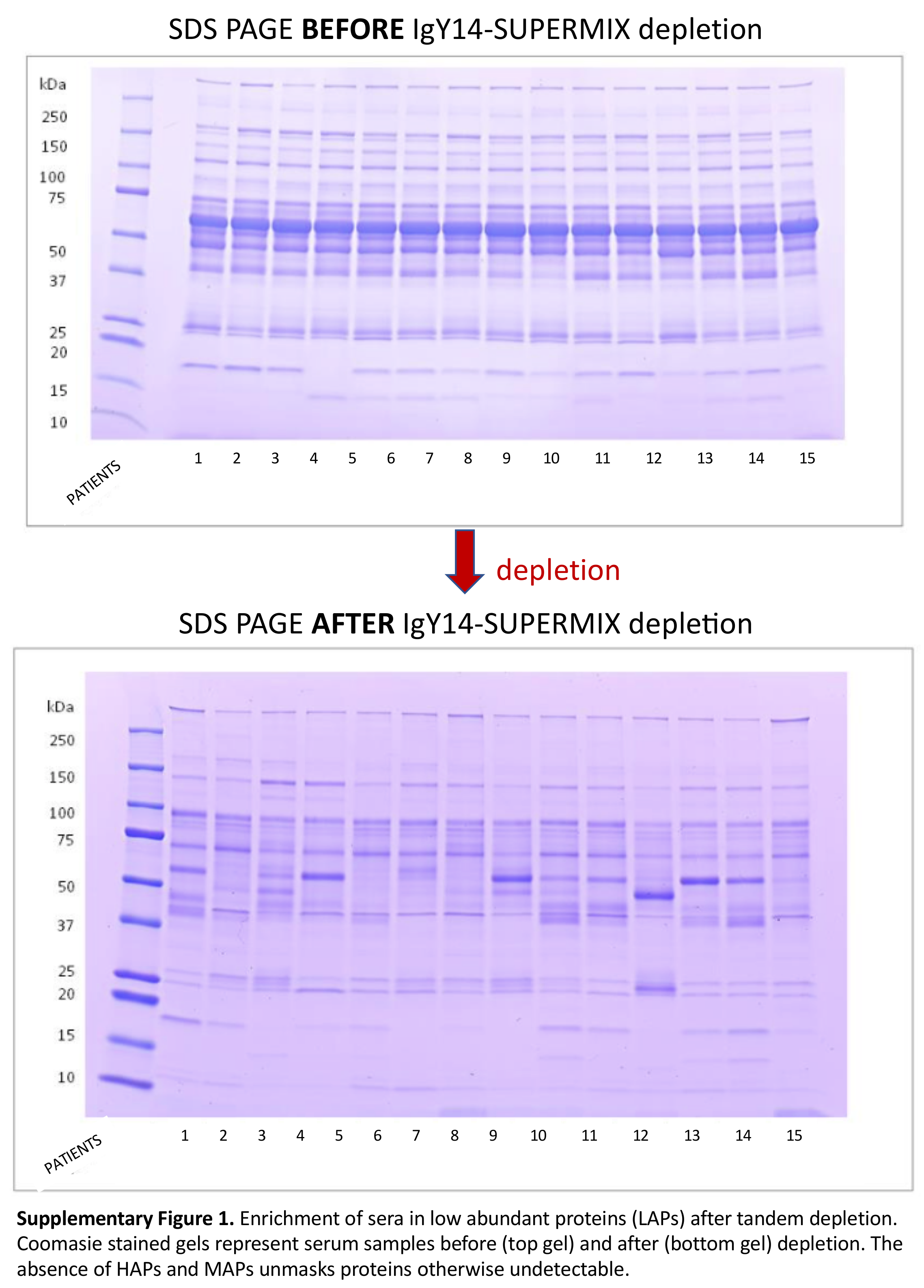

Supplement: Supplementary file 1 — Supplementary Material 1 [file 12967_2024_5344_MOESM1_ESM.png]
