# Supplementary material for: Untargeted blood serum proteomics identifies novel proteins related to neurological recovery after human spinal cord injury
Source: J Transl Med. 2024 Jul 17;22:666. doi: 10.1186/s12967-024-05344-y (PMC11256486; doi:10.1186/s12967-024-05344-y)
Supplement: Supplementary file 2 — Supplementary Material 2 [file 12967_2024_5344_MOESM2_ESM.docx]

**Table S1: Demographical characteristics of control individuals (Healthy Control and Spine fracture).**

|  | Healthy controls (HC) | Spine fracture (SPFC) |
| --- | --- | --- |
| Age (years)  Range  Mean ± sem | 24-73  52 ± 3.7 | 19-65  45 ± 10.5 |
| Sex (n)  Female  Male | 7  34 | 1  8 |
