# Supplementary material for: Untargeted blood serum proteomics identifies novel proteins related to neurological recovery after human spinal cord injury
Source: J Transl Med. 2024 Jul 17;22:666. doi: 10.1186/s12967-024-05344-y (PMC11256486; doi:10.1186/s12967-024-05344-y)
Supplement: Supplementary file 3 — Supplementary Material 3 [file 12967_2024_5344_MOESM3_ESM.docx]

Table S2. Proteins depleted by IgY14-Supermix sequential depletion (as specified by manufacturers and Shi et al., 2012)

| **High Abundant Proteins (HAP) depleted by IgY14 column** | | **Medium Abundant Proteins (MAP) depleted by**  **Supermix column** | |
| --- | --- | --- | --- |
| ***Gene name*** | ***Targeted Protein*** | ***Gene name*** | ***Targeted Protein*** |
| ALB | Albumin | A1BG | Alpha-1B-glycoprotein |
| IGHG | IgG | APCS | Serum amyloid P-component |
| SERPINA1 | Alpha-Antitrypsin | APOH | Beta-2-glycoprotein 1 |
| IGHA | IgA | C1QA | Complement C1Q subunit A |
| IGHM | IgM | C1QA | Complement C1Q subunit C |
| TF | Transferrin | C1QB | Complement C1Q subunit B |
| HP | Haptoglobulin | C3 | Complement C3 |
| FGG; FGA | Fibrinogen | C4A | Complement 4A |
| A2M | Alpha 2-Macroglobulin | C4B | Complement 4B |
| C3 | Complement C3 | C4BPA | C4B-binding protein alpha chain |
| ORM1, ORM2 | Orosomucoid | C4BPB | C4B-binding protein beta chain |
| APOA1 | Apolipoprotein A1 | C5 | Complement C5 |
| APOA2 | Apolipoprotein A2 | C6 | Complement component C6 |
| APOB | Apolipoprotein B | C7 | Complement component 7 |
|  |  | C8A | Complement C8 alpha chain |
|  |  | C8B | Complement C8 beta chain |
|  |  | C8G | Complement C8 gamma chain |
|  |  | CFB | Complement Factor B |
|  |  | CFH | Complement factor H |
|  |  | CFHR1 | Complement factor H-related protein 1 |
|  |  | CFHR2 | Complement factor H-related protein 2 |
|  |  | CFHR3 | Complement factor H-related protein 3 |
|  |  | CFHR5 | Complement factor H-related 5 |
|  |  | CFP | Properdin |
|  |  | CP | Ceruloplasmin |
|  |  | FGA | Fibrinogen alpha chain |
|  |  | FGB | Fibrinogen beta chain |
|  |  | FGG | Fibrinogen gamma chain |
|  |  | FN1 | Fibronectin |
|  |  | HPX | Hemopexin |
|  |  | HRG | Histidine-rich glycoprotein |
|  |  | ITIH1 | ITH1 |
|  |  | ITIH2 | ITH2 |
|  |  | ITIH4 | Inter-alpha-trypsin inhibitor heavy chain H4 |
|  |  | KNG1 | Isoform HMW of kininogen-1 |
|  |  | KNG1 | Isoform LMW of kininogen-1 |
|  |  | LOC441368 | Similar to ceruloplasmin |
|  |  | LPA | Apolipoprotein |
|  |  | PLG | Plasminogen |
|  |  | PROS1 | Vitamin K-dependent protein S |
|  |  | RBP4 | Plasma retinol-binding protein |
|  |  | SERPINC1 | Antithrombin III variant |
|  |  | SERPINC1 | SERPINC1 protein |
|  |  | VTN | Vitronectin |
