# Supplementary material for: Untargeted blood serum proteomics identifies novel proteins related to neurological recovery after human spinal cord injury
Source: J Transl Med. 2024 Jul 17;22:666. doi: 10.1186/s12967-024-05344-y (PMC11256486; doi:10.1186/s12967-024-05344-y)
Supplement: Supplementary file 5 — Supplementary Material 5 [file 12967_2024_5344_MOESM5_ESM.pdf]

HOSPITAL NACIONAL DE PARAPLÉJICOS Toledo

sescam Servicio de Salud de Castilla-La Mancha

Angel Arevalo-Martin
Hospital Nacional de Paraplejicos
Finca la Peraleda, s/n
45071 Toledo (Spain)
Office number: +34 925247718
E-mail: aarevalom@sescam.jccm.es

Dear Dr. Marincola,

On behalf of our group of authors, I would like to thank you for the opportunity to revise our manuscript for consideration to publish it at *Journal of Translational Medicine*.

We would also like to express our sincere gratitude to the reviewers for their highly insightful and constructive comments. We have prepared a revised version of the manuscript addressing the comments and suggestions from the reviewers.

Looking forward to hearing from you,

Yours sincerely,

Angel Arevalo-Martin
